# Bringing metabolic networks to life: integration of kinetic, metabolic, and proteomic data

**DOI:** 10.1186/1742-4682-3-42

**Published:** 2006-12-15

**Authors:** Wolfram Liebermeister, Edda Klipp

**Affiliations:** 1 Computational Systems Biology, Max Planck Institute for Molecular Genetics, Ihnestraße 63-73, 14195 Berlin, Germany

## Abstract

**Background:**

Translating a known metabolic network into a dynamic model requires reasonable guesses of all enzyme parameters. In Bayesian parameter estimation, model parameters are described by a posterior probability distribution, which scores the potential parameter sets, showing how well each of them agrees with the data and with the prior assumptions made.

**Results:**

We compute posterior distributions of kinetic parameters within a Bayesian framework, based on integration of kinetic, thermodynamic, metabolic, and proteomic data. The structure of the metabolic system (i.e., stoichiometries and enzyme regulation) needs to be known, and the reactions are modelled by convenience kinetics with thermodynamically independent parameters. The parameter posterior is computed in two separate steps: a first posterior summarises the available data on enzyme kinetic parameters; an improved second posterior is obtained by integrating metabolic fluxes, concentrations, and enzyme concentrations for one or more steady states. The data can be heterogenous, incomplete, and uncertain, and the posterior is approximated by a multivariate log-normal distribution. We apply the method to a model of the threonine synthesis pathway: the integration of metabolic data has little effect on the marginal posterior distributions of individual model parameters. Nevertheless, it leads to strong correlations between the parameters in the joint posterior distribution, which greatly improve the model predictions by the following Monte-Carlo simulations.

**Conclusion:**

We present a standardised method to translate metabolic networks into dynamic models. To determine the model parameters, evidence from various experimental data is combined and weighted using Bayesian parameter estimation. The resulting posterior parameter distribution describes a statistical ensemble of parameter sets; the parameter variances and correlations can account for missing knowledge, measurement uncertainties, or biological variability. The posterior distribution can be used to sample model instances and to obtain probabilistic statements about the model's dynamic behaviour.

## Background

### Dynamic simulation of metabolic systems

Local perturbations of biochemical systems, e.g. by differential gene expression or drug treatment, can lead to global effects that are by no means self-evident. An intention of systems biology is to predict them by computer simulations, which requires mathematical models of the biochemical networks. The structure of metabolic networks has been characterised for many organisms [1-3], and metabolic fluxes in large networks [4-6] are successfully described by pathway- or constraint-based methods [7-10]. However, such methods do not explain how the fluxes are actually evoked by the activities of enzymes and how they respond to moderate perturbations.

These questions can be answered by kinetic models, which employ differential equations to describe the temporal behaviour of the system. Kinetic models allow for bifurcation and control analysis [11-13]; parameter distributions [14-17] can be used to explore their variability and potential behaviour. Unfortunately, there is a disproportion between the high number of parameters contained in kinetic models and the relatively incomplete data available: kinetic laws are not known for most enzymes, and kinetic and metabolic data are sparse, uncertain, and dispersed over databases [18-20], models [21,22], and the literature [23,24]. Therefore, parameter estimation is an integral part of kinetic modelling, and model fitting is currently receiving increasing attention [25-29].

Interestingly, some dynamic properties are determined by the network structure alone, for instance, the sums of metabolic control coefficients described in summation theorems; other properties may be rather insensitive to the choice of parameters. Parameter ensembles [15,30] can be used to assess and distinguish the respective impact of structure and kinetics. Given a metabolic network, it would be desirable at least to know plausible ranges and correlations for all model parameters, in agreement with the known data. Here we suggest a way to achieve this by collecting and integrating heterogenous data in an automatic manner.

### Outline of the paper

**Figure 1:**
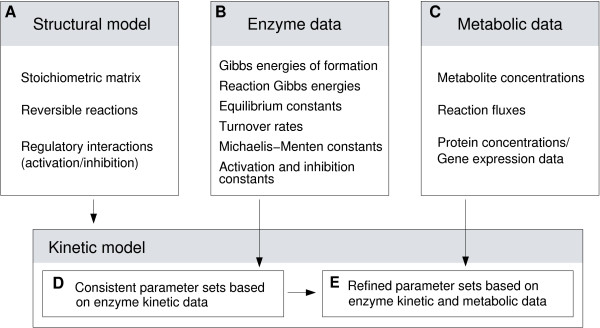
**Data integration pipeline**. A metabolic network (A) is translated into a kinetic model. The model parameters are described by statistical distributions. Experimental values of enzyme parameters (B) are used to obtain a first, kinetics-based distribution of enzyme parameters (D). A fit to metabolic data (C) such as metabolite and enzyme concentrations and metabolic fluxes leads to a second, metabolics-based, distribution of system parameters (thermodynamic and kinetic parameters) and state parameters (metabolite and enzyme concentrations) (E). The system parameters describe the enzymatic reactions in general and remain constant for a given cell; fluxes and concentrations can fluctuate and depend on specific states of the cell; however, integrating metabolic data from several experiments can also improve the fit of kinetic parameters.

We aim at translating a metabolic network into a kinetic model, using the convenience kinetics described in the companion article [31]. For parameter estimation, we use as many data as possible: besides thermodynamic and kinetic parameters, we also integrate proteome data and metabolic concentrations and fluxes (see Figure 1).

As the data are incomplete and unreliable, we do not describe the model parameters by sharp values, but by a joint posterior distribution [15]. Even if the data do not suffice for an exact parameter fit, we will still obtain a model; the uncertainty of the parameters and correlations between them can be read directly from the posterior parameter distribution. The posterior summarises all information that has been put into the model and can be used to provide parameter ranges for further modelling, to sample model instances [30,32], or to predict confidence intervals of steady state fluxes and concentrations or responses to differential expression [15]. We illustrate the approach by estimating parameters for the threonine pathway in E. coli [33]. A list of symbols and a description of the estimation algorithm is provided [See Additional file 1].

### Kinetic models with convenience kinetics

Let us first introduce some notation for kinetic modelling. In the setting of deterministic differential equations, the concentrations of substances in a biochemical system follow the balance equations

ddtc=N v(c,k).     (1)
 MathType@MTEF@5@5@+=feaafiart1ev1aaatCvAUfKttLearuWrP9MDH5MBPbIqV92AaeXatLxBI9gBaebbnrfifHhDYfgasaacH8akY=wiFfYdH8Gipec8Eeeu0xXdbba9frFj0=OqFfea0dXdd9vqai=hGuQ8kuc9pgc9s8qqaq=dirpe0xb9q8qiLsFr0=vr0=vr0dc8meaabaqaciaacaGaaeqabaqabeGadaaakeaadaWcaaqaaiabbsgaKbqaaiabbsgaKjabdsha0baacqWGJbWycqGH9aqpcqWGobGtcqqGGaaicqWG2bGDcqGGOaakcqWGJbWycqGGSaalcqWGRbWAcqGGPaqkcqGGUaGlcaWLjaGaaCzcamaabmaabaGaeGymaedacaGLOaGaayzkaaaaaa@4062@

The vectors *c*, *v*, and *k *contain the metabolite concentrations, the reaction velocities, and (non-logarithmic) system parameters, respectively. Some of the metabolites may be considered external or buffered; in the model, their concentrations are fixed values contained in the parameter vector *k*. Concentrations are measured in mM, time in seconds, energies in J/mol.

In a stationary state, all metabolite concentrations remain constant over time: by solving 0 = *Nv*(*c, k*) for the concentration vector *c *at given parameters *k*, we obtain the steady-state state concentrations *s*(*k*). The corresponding reaction velocities *j*(*k*) = *v*(*s*(*k*), *k*) are called stationary fluxes. The response of steady state variables *y*(*k*) (which may be concentrations *s*(*k*), fluxes *j*(*k*), or functions thereof) to small parameter changes is described by the response coefficients R^imY
 MathType@MTEF@5@5@+=feaafiart1ev1aaatCvAUfKttLearuWrP9MDH5MBPbIqV92AaeXatLxBI9gBaebbnrfifHhDYfgasaacH8akY=wiFfYdH8Gipec8Eeeu0xXdbba9frFj0=OqFfea0dXdd9vqai=hGuQ8kuc9pgc9s8qqaq=dirpe0xb9q8qiLsFr0=vr0=vr0dc8meaabaqaciaacaGaaeqabaqabeGadaaakeaacuWGsbGugaqcamaaDaaaleaacqWGPbqAcqWGTbqBaeaacqWGzbqwaaaaaa@320F@ = ∂*y*_*i*_/∂*k*_*m*_. They can be computed efficiently [13,34] if the steady state is known. The relationships between logarithmic parameters *θ*_*m *_= In *k*_*m *_and non-logarithmic variables *y*_*i *_are described by right-normalised response coefficients or sensitivities Rθmyi
 MathType@MTEF@5@5@+=feaafiart1ev1aaatCvAUfKttLearuWrP9MDH5MBPbIqV92AaeXatLxBI9gBaebbnrfifHhDYfgasaacH8akY=wiFfYdH8Gipec8Eeeu0xXdbba9frFj0=OqFfea0dXdd9vqai=hGuQ8kuc9pgc9s8qqaq=dirpe0xb9q8qiLsFr0=vr0=vr0dc8meaabaqaciaacaGaaeqabaqabeGadaaakeaacqWGsbGudaqhaaWcbaacciGae8hUde3aaSbaaWqaaiabd2gaTbqabaaaleaacqWG5bqEdaWgaaadbaGaemyAaKgabeaaaaaaaa@3461@ = ∂*y*_*i*_/∂*θ*_*m *_= *k*_*m *_∂*y*_*i*_/∂*k*_*m*_.

The dynamic behaviour of a model depends strongly on the rate laws *v*(·) that are used in the system equations (1). Here we use the convenience kinetics, a versatile and relatively simple rate law described in the companion article [31]. A metabolic model with convenience kinetics is characterised by the following system parameters: (i) an energy constant kiG
 MathType@MTEF@5@5@+=feaafiart1ev1aaatCvAUfKttLearuWrP9MDH5MBPbIqV92AaeXatLxBI9gBaebbnrfifHhDYfgasaacH8akY=wiFfYdH8Gipec8Eeeu0xXdbba9frFj0=OqFfea0dXdd9vqai=hGuQ8kuc9pgc9s8qqaq=dirpe0xb9q8qiLsFr0=vr0=vr0dc8meaabaqaciaacaGaaeqabaqabeGadaaakeaacqWGRbWAdaqhaaWcbaGaemyAaKgabaacbaGae83raCeaaaaa@30AF@ (dimensionless) for each metabolite *i*; (ii) a velocity constant klV
 MathType@MTEF@5@5@+=feaafiart1ev1aaatCvAUfKttLearuWrP9MDH5MBPbIqV92AaeXatLxBI9gBaebbnrfifHhDYfgasaacH8akY=wiFfYdH8Gipec8Eeeu0xXdbba9frFj0=OqFfea0dXdd9vqai=hGuQ8kuc9pgc9s8qqaq=dirpe0xb9q8qiLsFr0=vr0=vr0dc8meaabaqaciaacaGaaeqabaqabeGadaaakeaacqWGRbWAdaqhaaWcbaGaemiBaWgabaacbaGae8Nvayfaaaaa@30D3@ (1/s) for each reaction *l*; (iii) a reactant constant kliM
 MathType@MTEF@5@5@+=feaafiart1ev1aaatCvAUfKttLearuWrP9MDH5MBPbIqV92AaeXatLxBI9gBaebbnrfifHhDYfgasaacH8akY=wiFfYdH8Gipec8Eeeu0xXdbba9frFj0=OqFfea0dXdd9vqai=hGuQ8kuc9pgc9s8qqaq=dirpe0xb9q8qiLsFr0=vr0=vr0dc8meaabaqaciaacaGaaeqabaqabeGadaaakeaacqWGRbWAdaqhaaWcbaGaemiBaWMaemyAaKgabaacbaGae8xta0eaaaaa@321C@ (mM) for each substrate or product *i *of a reaction *l*; and (iv) an activation or inhibition constant kliA
 MathType@MTEF@5@5@+=feaafiart1ev1aaatCvAUfKttLearuWrP9MDH5MBPbIqV92AaeXatLxBI9gBaebbnrfifHhDYfgasaacH8akY=wiFfYdH8Gipec8Eeeu0xXdbba9frFj0=OqFfea0dXdd9vqai=hGuQ8kuc9pgc9s8qqaq=dirpe0xb9q8qiLsFr0=vr0=vr0dc8meaabaqaciaacaGaaeqabaqabeGadaaakeaacqWGRbWAdaqhaaWcbaGaemiBaWMaemyAaKgabaacbaGae8xqaeeaaaaa@3204@ or kliI
 MathType@MTEF@5@5@+=feaafiart1ev1aaatCvAUfKttLearuWrP9MDH5MBPbIqV92AaeXatLxBI9gBaebbnrfifHhDYfgasaacH8akY=wiFfYdH8Gipec8Eeeu0xXdbba9frFj0=OqFfea0dXdd9vqai=hGuQ8kuc9pgc9s8qqaq=dirpe0xb9q8qiLsFr0=vr0=vr0dc8meaabaqaciaacaGaaeqabaqabeGadaaakeaacqWGRbWAdaqhaaWcbaGaemiBaWMaemyAaKgabaacbaGae8xsaKeaaaaa@3214@ (mM) for each metabolite *i *that regulates a reaction *l*.

The mathematical form of the convenience rate law depends on the reaction stoichiometry: for a chemical reaction A + B → P + Q without activators and inhibitors and with enzyme concentration *E*, it reads

v(a,b,p,q)=Ek+cata˜b˜−k−catp˜q˜1+a˜+b˜+a˜b˜+p˜+q˜+p˜q˜,     (2)
 MathType@MTEF@5@5@+=feaafiart1ev1aaatCvAUfKttLearuWrP9MDH5MBPbIqV92AaeXatLxBI9gBaebbnrfifHhDYfgasaacH8akY=wiFfYdH8Gipec8Eeeu0xXdbba9frFj0=OqFfea0dXdd9vqai=hGuQ8kuc9pgc9s8qqaq=dirpe0xb9q8qiLsFr0=vr0=vr0dc8meaabaqaciaacaGaaeqabaqabeGadaaakeaacqWG2bGDcqGGOaakcqWGHbqycqGGSaalcqWGIbGycqGGSaalcqWGWbaCcqGGSaalcqWGXbqCcqGGPaqkcqGH9aqpcqWGfbqrdaWcaaqaaiabdUgaRnaaDaaaleaacqGHRaWkaeaaieaacqWFJbWycqWFHbqycqWF0baDaaGccuWGHbqygaacaiqbdkgaIzaaiaGaeyOeI0Iaem4AaS2aa0baaSqaaiabgkHiTaqaaiab=ngaJjab=fgaHjab=rha0baakiqbdchaWzaaiaGafmyCaeNbaGaaaeaacqaIXaqmcqGHRaWkcuWGHbqygaacaiabgUcaRiqbdkgaIzaaiaGaey4kaSIafmyyaeMbaGaacuWGIbGygaacaiabgUcaRiqbdchaWzaaiaGaey4kaSIafmyCaeNbaGaacqGHRaWkcuWGWbaCgaacaiqbdghaXzaaiaaaaiabcYcaSiaaxMaacaWLjaWaaeWaaeaacqaIYaGmaiaawIcacaGLPaaaaaa@63CA@

where *ã *= *a*/kAM
 MathType@MTEF@5@5@+=feaafiart1ev1aaatCvAUfKttLearuWrP9MDH5MBPbIqV92AaeXatLxBI9gBaebbnrfifHhDYfgasaacH8akY=wiFfYdH8Gipec8Eeeu0xXdbba9frFj0=OqFfea0dXdd9vqai=hGuQ8kuc9pgc9s8qqaq=dirpe0xb9q8qiLsFr0=vr0=vr0dc8meaabaqaciaacaGaaeqabaqabeGadaaakeaacqWGRbWAdaqhaaWcbaacbaGae8xqaeeabaGae8xta0eaaaaa@3067@; normalised concentrations for the other reactants are defined accordingly. The turnover rates read

k±cat=kV(kAGkAMkBGkBMkPGkPMkQGkQM)±1/2.     (3)
 MathType@MTEF@5@5@+=feaafiart1ev1aaatCvAUfKttLearuWrP9MDH5MBPbIqV92AaeXatLxBI9gBaebbnrfifHhDYfgasaacH8akY=wiFfYdH8Gipec8Eeeu0xXdbba9frFj0=OqFfea0dXdd9vqai=hGuQ8kuc9pgc9s8qqaq=dirpe0xb9q8qiLsFr0=vr0=vr0dc8meaabaqaciaacaGaaeqabaqabeGadaaakeaacqWGRbWAdaqhaaWcbaGaeyySaelabaacbaGae83yamMae8xyaeMae8hDaqhaaOGaeyypa0Jaem4AaS2aaWbaaSqabeaacqWFwbGvaaGcdaqadaqaamaalaaabaGaem4AaS2aa0baaSqaaiab=feabbqaaiab=DeahbaakiabdUgaRnaaDaaaleaacqWFbbqqaeaacqWFnbqtaaGccqWGRbWAdaqhaaWcbaGae8NqaieabaGae83raCeaaOGaem4AaS2aa0baaSqaaiab=jeacbqaaiab=1eanbaaaOqaaiabdUgaRnaaDaaaleaacqWFqbauaeaacqWFhbWraaGccqWGRbWAdaqhaaWcbaGae8huaafabaGae8xta0eaaOGaem4AaS2aa0baaSqaaiab=ffarbqaaiab=DeahbaakiabdUgaRnaaDaaaleaacqWFrbquaeaacqWFnbqtaaaaaaGccaGLOaGaayzkaaWaaWbaaSqabeaacqGHXcqScqaIXaqmcqGGVaWlcqaIYaGmaaGccqGGUaGlcaWLjaGaaCzcamaabmaabaGaeG4mamdacaGLOaGaayzkaaaaaa@6160@

This parametrisation of the rate law ensures that any combination of positive parameter values is thermodynamically feasible.

## Method

### Parameter estimation

Bayesian parameter estimation [35] integrates two sources of knowledge: (i) expectations about the model parameters are quantified by a prior probability density *p*(*θ*). The prior can describe typical parameter ranges or summarise the results of earlier experiments; (ii) the support by experimental data is quantified by the likelihood function *p*(x*|*θ*). By combining both kinds of information, we can obtain a posterior distribution, which describes how plausible certain parameter sets appear, taking into account both the prior information and the experimental data.

In our case, the logarithmic values of all system parameters are collected in a vector *θ*^kin^. To model cells in specific experimental situations, we specify additional state parameters: a specific steady state *m *is characterised by enzyme concentrations El(m)
 MathType@MTEF@5@5@+=feaafiart1ev1aaatCvAUfKttLearuWrP9MDH5MBPbIqV92AaeXatLxBI9gBaebbnrfifHhDYfgasaacH8akY=wiFfYdH8Gipec8Eeeu0xXdbba9frFj0=OqFfea0dXdd9vqai=hGuQ8kuc9pgc9s8qqaq=dirpe0xb9q8qiLsFr0=vr0=vr0dc8meaabaqaciaacaGaaeqabaqabeGadaaakeaacqWGfbqrdaqhaaWcbaGaemiBaWgabaGaeiikaGIaemyBa0MaeiykaKcaaaaa@3262@ and fixed concentrations si(m)
 MathType@MTEF@5@5@+=feaafiart1ev1aaatCvAUfKttLearuWrP9MDH5MBPbIqV92AaeXatLxBI9gBaebbnrfifHhDYfgasaacH8akY=wiFfYdH8Gipec8Eeeu0xXdbba9frFj0=OqFfea0dXdd9vqai=hGuQ8kuc9pgc9s8qqaq=dirpe0xb9q8qiLsFr0=vr0=vr0dc8meaabaqaciaacaGaaeqabaqabeGadaaakeaacqWGZbWCdaqhaaWcbaGaemyAaKgabaGaeiikaGIaemyBa0MaeiykaKcaaaaa@32B8@ for the external metabolites. Again, we collect all logarithmic values in a vector *θ*^met^, and we define the parameter vector *θ *= (*θ*^kin^, *θ*^met^). Variable metabolites and metabolic fluxes are not treated as state parameters, but computed from the parameters via the steady-state equation.

The parameter estimation proceeds in two steps: in the first step, only the system parameters are fitted to thermodynamic and kinetic data, such as Gibbs free energies of formation, reaction Gibbs free energies, equilibrium constants, *k*^M ^values, *k*^I ^values, *k*^A ^values, and turnover rates. The logarithms of the experimental values are collected in a large vector x*. With the convenience kinetics, the corresponding vector *x *of model predictions is a linear function of *θ*^kin^, which greatly simplifies the calculation [31]. In the second step, the parameter estimates are further improved by a fit to metabolite concentrations, metabolic fluxes, and protein concentrations from one or more steady states; we shall summarize them here as "metabolic data" and collect them in a vector y*. The posterior from the first step is used as a prior in the second step: therefore, no information from the first step will be lost.

**Figure 2:**
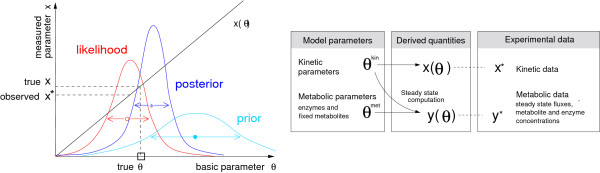
**Bayesian parameter estimation**. Left: a kinetic parameter *θ *(abscissa) determines an observed parameter *x *(ordinate). Adding Gaussian noise to the true value *x *yields the experimental value x*, which then gives rise to a likelihood function *p*(x*|*θ*) (red). Prior distribution *p*(*θ*) (light blue) and likelihood function lead to a posterior distribution *p*(*θ*|x*)(dark blue), which represents a refined estimate of the original parameter. Right: parameters and data determine the likelihood function for a metabolic network model. Each set of system parameters *θ*^kin ^and state parameters *θ*^met ^(left) will lead to predictions *x *and *y *of the observable quantities (centre), which can be compared to the corresponding experimental values x* and y* (right).

The way from prior to posterior distribution is shown in Figure 2. According to the Bayes formula [35], the posterior probability density *p*(*θ*|x*, y*) of the model parameters *θ *given the experimental data x* and y* can be computed from the prior probability density *p*(*θ*) and from the likelihood function *p*(x*|*θ*):

*p*(*θ*|x*, y*) ~ *p*(x*, y*|*θ*) *p*(*θ*)

= *p*(y*|*θ*) *p*(x*|*θ*) *p*(*θ*).     (4)

### Prior and likelihood function

The posterior depends on the prior and the likelihood function; for our metabolic networks, we specify them as follows:

1. The prior distribution of *θ *is a multivariate Gaussian distribution N
 MathType@MTEF@5@5@+=feaafiart1ev1aaatCvAUfKttLearuWrP9MDH5MBPbIqV92AaeXatLxBI9gBamrtHrhAL1wy0L2yHvtyaeHbnfgDOvwBHrxAJfwnaebbnrfifHhDYfgasaacH8akY=wiFfYdH8Gipec8Eeeu0xXdbba9frFj0=OqFfea0dXdd9vqai=hGuQ8kuc9pgc9s8qqaq=dirpe0xb9q8qiLsFr0=vr0=vr0dc8meaabaqaciaacaGaaeqabaWaaeGaeaaakeaaimaacqWFneVtaaa@383B@, that is,

*θ *= N
 MathType@MTEF@5@5@+=feaafiart1ev1aaatCvAUfKttLearuWrP9MDH5MBPbIqV92AaeXatLxBI9gBamrtHrhAL1wy0L2yHvtyaeHbnfgDOvwBHrxAJfwnaebbnrfifHhDYfgasaacH8akY=wiFfYdH8Gipec8Eeeu0xXdbba9frFj0=OqFfea0dXdd9vqai=hGuQ8kuc9pgc9s8qqaq=dirpe0xb9q8qiLsFr0=vr0=vr0dc8meaabaqaciaacaGaaeqabaWaaeGaeaaakeaaimaacqWFneVtaaa@383B@(θ¯
 MathType@MTEF@5@5@+=feaafiart1ev1aaatCvAUfKttLearuWrP9MDH5MBPbIqV92AaeXatLxBI9gBaebbnrfifHhDYfgasaacH8akY=wiFfYdH8Gipec8Eeeu0xXdbba9frFj0=OqFfea0dXdd9vqai=hGuQ8kuc9pgc9s8qqaq=dirpe0xb9q8qiLsFr0=vr0=vr0dc8meaabaqaciaacaGaaeqabaqabeGadaaakeaaiiGacuWF4oqCgaqeaaaa@2E81@_(0)_, *C*_(0)_)     (5)

with probability density *p*(*θ*), mean vector θ¯
 MathType@MTEF@5@5@+=feaafiart1ev1aaatCvAUfKttLearuWrP9MDH5MBPbIqV92AaeXatLxBI9gBaebbnrfifHhDYfgasaacH8akY=wiFfYdH8Gipec8Eeeu0xXdbba9frFj0=OqFfea0dXdd9vqai=hGuQ8kuc9pgc9s8qqaq=dirpe0xb9q8qiLsFr0=vr0=vr0dc8meaabaqaciaacaGaaeqabaqabeGadaaakeaaiiGacuWF4oqCgaqeaaaa@2E81@_(0)_, and a diagonal covariance matrix *C*_(0)_. Mean and variance of each single parameter are chosen depending on the parameter type (that is, different distributions for energy constants, *k*^M ^values, and so on). Prior distributions for the different parameter types can be derived from empirical distributions of parameter values. The values found in databases and the literature (see table 1) typically span several orders of magnitude.

**Table 1:** Empirical parameter ranges

Parameter		⟨*x*⟩	*σ*_*x*_		e^⟨*x*⟩^	eσx MathType@MTEF@5@5@+=feaafiart1ev1aaatCvAUfKttLearuWrP9MDH5MBPbIqV92AaeXatLxBI9gBaebbnrfifHhDYfgasaacH8akY=wiFfYdH8Gipec8Eeeu0xXdbba9frFj0=OqFfea0dXdd9vqai=hGuQ8kuc9pgc9s8qqaq=dirpe0xb9q8qiLsFr0=vr0=vr0dc8meaabaqaciaacaGaaeqabaqabeGadaaakeaacqWGLbqzdaahaaWcbeqaaGGaciab=n8aZnaaBaaameaacqWG4baEaeqaaaaaaaa@319C@	# samples	ref.
Turnover rate	*k*^cat^	1.95	3.3	7.0	s^-1^	27.1	7559	[18]
Substrate constant	*k*^M^	-1.77	3.0	0.17	mM	20.1	44766	[18]
Inhibition constant	*k*^I^	-2.81	4.1	0.06	mM	60.3	4338	[18]
Energy constant	*k*^G^	-0.24	0.18	0.79		1.2	142	[23]
Equilibrium constant	*k*^eq^	-	5.4	-		212	1309	[19]
Protein molecules/cell		7.82	1.56	2480		4.7	3868	[20]
Protein concentration	*E*_*l*_	-10.23	1.56	3.6·10^-5^	mM	4.7	3868	[20]
Metab. concentration	c_i_	-1.97	1.94	0.14	mM	7.0	49	[24]

Typical ranges of system parameters (top) and state parameters (bottom). Different types of parameters show specific mean values and standard deviations. Energy constants were predicted from the molecule structures, all other data were obtained from experiments. Numbers of protein molecules were measured in the yeast *S. cerevisiae*. The symbols ⟨*x*⟩ and *σ*_*x *_denote mean values and standard deviations of the natural logarithms, in data sets of different sizes ("# samples"). These values can be used to predefine a prior distribution for model parameters. The exponential values exp(⟨*x*⟩) and exp(*σ*_*x*_) denote, respectively, the geometric mean and a typical uncertainty factor of the parameter type.

2. The likelihood functions *p*(y*|*θ*) and *p*(x*|*θ*) represent a simple model of the measurement process: we assume that the experimental values x* and y* equal the values predicted by the model plus uncorrelated additive Gaussian noise, hence

x* = N
 MathType@MTEF@5@5@+=feaafiart1ev1aaatCvAUfKttLearuWrP9MDH5MBPbIqV92AaeXatLxBI9gBamrtHrhAL1wy0L2yHvtyaeHbnfgDOvwBHrxAJfwnaebbnrfifHhDYfgasaacH8akY=wiFfYdH8Gipec8Eeeu0xXdbba9frFj0=OqFfea0dXdd9vqai=hGuQ8kuc9pgc9s8qqaq=dirpe0xb9q8qiLsFr0=vr0=vr0dc8meaabaqaciaacaGaaeqabaWaaeGaeaaakeaaimaacqWFneVtaaa@383B@(*x*(*θ*), *C*_x_)     (6)

y* = N
 MathType@MTEF@5@5@+=feaafiart1ev1aaatCvAUfKttLearuWrP9MDH5MBPbIqV92AaeXatLxBI9gBamrtHrhAL1wy0L2yHvtyaeHbnfgDOvwBHrxAJfwnaebbnrfifHhDYfgasaacH8akY=wiFfYdH8Gipec8Eeeu0xXdbba9frFj0=OqFfea0dXdd9vqai=hGuQ8kuc9pgc9s8qqaq=dirpe0xb9q8qiLsFr0=vr0=vr0dc8meaabaqaciaacaGaaeqabaWaaeGaeaaakeaaimaacqWFneVtaaa@383B@(*y*(*θ*), *C*_y_).     (7)

We assume diagonal covariance matrices *C*_x _= diag(*σ*_x_)^2 ^and *C*_y _= diag(*σ*_y_)^2^, where the vectors *σ*_x _and *σ*_y _contain noise levels for each single measurement.

To establish the likelihood functions (6) and (7), the kinetic parameters *x *and the metabolic data *y *have to be expressed as functions of the model parameters *θ *(see Figure 2, right). The logarithmic parameters in the convenience rate law fulfil a linear relationship [31].

*x*(*θ*) = Rθx
 MathType@MTEF@5@5@+=feaafiart1ev1aaatCvAUfKttLearuWrP9MDH5MBPbIqV92AaeXatLxBI9gBaebbnrfifHhDYfgasaacH8akY=wiFfYdH8Gipec8Eeeu0xXdbba9frFj0=OqFfea0dXdd9vqai=hGuQ8kuc9pgc9s8qqaq=dirpe0xb9q8qiLsFr0=vr0=vr0dc8meaabaqaciaacaGaaeqabaqabeGadaaakeaacqWGsbGudaqhaaWcbaacciGae8hUdehabaGaemiEaGhaaaaa@313C@*θ *    (8)

with a sparse sensitivity matrix Rθx
 MathType@MTEF@5@5@+=feaafiart1ev1aaatCvAUfKttLearuWrP9MDH5MBPbIqV92AaeXatLxBI9gBaebbnrfifHhDYfgasaacH8akY=wiFfYdH8Gipec8Eeeu0xXdbba9frFj0=OqFfea0dXdd9vqai=hGuQ8kuc9pgc9s8qqaq=dirpe0xb9q8qiLsFr0=vr0=vr0dc8meaabaqaciaacaGaaeqabaqabeGadaaakeaacqWGsbGudaqhaaWcbaacciGae8hUdehabaGaemiEaGhaaaaa@313C@. A sensitivity matrix Rθx
 MathType@MTEF@5@5@+=feaafiart1ev1aaatCvAUfKttLearuWrP9MDH5MBPbIqV92AaeXatLxBI9gBaebbnrfifHhDYfgasaacH8akY=wiFfYdH8Gipec8Eeeu0xXdbba9frFj0=OqFfea0dXdd9vqai=hGuQ8kuc9pgc9s8qqaq=dirpe0xb9q8qiLsFr0=vr0=vr0dc8meaabaqaciaacaGaaeqabaqabeGadaaakeaacqWGsbGudaqhaaWcbaacciGae8hUdehabaGaemiEaGhaaaaa@313C@ related only to the kinetic parameters *θ*^kin ^can be constructed easily from the metabolic network [31]. The full Rθx
 MathType@MTEF@5@5@+=feaafiart1ev1aaatCvAUfKttLearuWrP9MDH5MBPbIqV92AaeXatLxBI9gBaebbnrfifHhDYfgasaacH8akY=wiFfYdH8Gipec8Eeeu0xXdbba9frFj0=OqFfea0dXdd9vqai=hGuQ8kuc9pgc9s8qqaq=dirpe0xb9q8qiLsFr0=vr0=vr0dc8meaabaqaciaacaGaaeqabaqabeGadaaakeaacqWGsbGudaqhaaWcbaacciGae8hUdehabaGaemiEaGhaaaaa@313C@ contains additional empty columns to account for the state parameters, which do not play a role for the computation of *x*. The concentrations of proteins and fixed metabolites follow trivially from the respective model parameters in *θ*; the metabolic concentrations and fluxes contained in *y*(*θ*) are computed numerically by solving the steady state equations.

### Computing the posterior distribution

Theoretically, we can obtain the posterior distribution *p*(*θ*|x*, y*) by inserting the distributions (5), (6), and (7) into (4). But how can we actually compute it? Standard methods for sampling the posterior distribution, such as Gibbs sampling [35], become unfeasible if the number of parameters is large. Therefore, we shall approximate the posterior by a Gaussian distribution around a local maximum of the posterior, the so-called posterior mode.

We proceed in two steps, first using the kinetic information and later adding the metabolic data. Instead of *p*(*θ*|x*, y*) itself, let us consider the function

F(θ)=−2ln⁡p(θ|x*,y*)=(θ−θ¯(0))TC(0)−1(θ−θ¯(0))+(x*−x(θ))TCx−1(x*−x(θ))+(y*−y(θ))TCy−1(y*−y(θ))+const.     (9)
 MathType@MTEF@5@5@+=feaafiart1ev1aaatCvAUfKttLearuWrP9MDH5MBPbIqV92AaeXatLxBI9gBaebbnrfifHhDYfgasaacH8akY=wiFfYdH8Gipec8Eeeu0xXdbba9frFj0=OqFfea0dXdd9vqai=hGuQ8kuc9pgc9s8qqaq=dirpe0xb9q8qiLsFr0=vr0=vr0dc8meaabaqaciaacaGaaeqabaqabeGadaaakeaafaqaaeWadaaabaGaemOrayKaeiikaGccciGae8hUdeNaeiykaKcabaGaeyypa0dabaGaeyOeI0IaeGOmaiJagiiBaWMaeiOBa4MaemiCaaNaeiikaGIae8hUdeNaeiiFaWNaemiEaGNaeiOkaOIaeiilaWIaemyEaKNaeiOkaOIaeiykaKcabaaabaGaeyypa0dabaGaeiikaGIae8hUdeNaeyOeI0Iaf8hUdeNbaebadaWgaaWcbaGaeiikaGIaeGimaaJaeiykaKcabeaakiabcMcaPmaaCaaaleqabaacbaGae4hvaqfaaOGaem4qam0aa0baaSqaaiabcIcaOiabicdaWiabcMcaPaqaaiabgkHiTiabigdaXaaakiabcIcaOiab=H7aXjabgkHiTiqb=H7aXzaaraWaaSbaaSqaaiabcIcaOiabicdaWiabcMcaPaqabaGccqGGPaqkcqGHRaWkcqGGOaakcqWG4baEcqGGQaGkcqGHsislcqWG4baEcqGGOaakcqWF4oqCcqGGPaqkcqGGPaqkdaahaaWcbeqaaiab+rfaubaakiabdoeadnaaDaaaleaacqGF4baEaeaacqGHsislcqaIXaqmaaGccqGGOaakcqWG4baEcqGGQaGkcqGHsislcqWG4baEcqGGOaakcqWF4oqCcqGGPaqkcqGGPaqkaeaaaeaaaeaacqGHRaWkcqGGOaakcqWG5bqEcqGGQaGkcqGHsislcqWG5bqEcqGGOaakcqWF4oqCcqGGPaqkcqGGPaqkdaahaaWcbeqaaiab+rfaubaakiabdoeadnaaDaaaleaacqqG5bqEaeaacqGHsislcqaIXaqmaaGccqGGOaakcqWG5bqEcqGGQaGkcqGHsislcqWG5bqEcqGGOaakcqWF4oqCcqGGPaqkcqGGPaqkcqGHRaWkcqGFJbWycqGFVbWBcqGFUbGBcqGFZbWCcqGF0baDcqGGUaGlaaGaaCzcaiaaxMaadaqadaqaaiabiMda5aGaayjkaiaawMcaaaaa@9E56@

If *F*(*θ*) is a quadratic function, the posterior is a Gaussian distribution. This is indeed the case as long as no metabolic data y* are considered: as *x*(*θ*) is linear, the first two terms are quadratic in *θ *and the corresponding posterior is Gaussian. We shall call it the first, or kinetics-based, posterior.

### Kinetics-based posterior

In the first step, we consider only measured kinetic parameters x*. The third term in (9) is neglected, and the posterior probability density reads *p*(*θ*|x*) ~ *p*(x*|*θ*) *p*(*θ*). The distribution is multivariate Gaussian N
 MathType@MTEF@5@5@+=feaafiart1ev1aaatCvAUfKttLearuWrP9MDH5MBPbIqV92AaeXatLxBI9gBamrtHrhAL1wy0L2yHvtyaeHbnfgDOvwBHrxAJfwnaebbnrfifHhDYfgasaacH8akY=wiFfYdH8Gipec8Eeeu0xXdbba9frFj0=OqFfea0dXdd9vqai=hGuQ8kuc9pgc9s8qqaq=dirpe0xb9q8qiLsFr0=vr0=vr0dc8meaabaqaciaacaGaaeqabaWaaeGaeaaakeaaimaacqWFneVtaaa@383B@(θ¯
 MathType@MTEF@5@5@+=feaafiart1ev1aaatCvAUfKttLearuWrP9MDH5MBPbIqV92AaeXatLxBI9gBaebbnrfifHhDYfgasaacH8akY=wiFfYdH8Gipec8Eeeu0xXdbba9frFj0=OqFfea0dXdd9vqai=hGuQ8kuc9pgc9s8qqaq=dirpe0xb9q8qiLsFr0=vr0=vr0dc8meaabaqaciaacaGaaeqabaqabeGadaaakeaaiiGacuWF4oqCgaqeaaaa@2E81@_(1)_, *C*_(1)_) with mean and covariance matrix (see [35])

θ¯(1)=(C(0)−1+(Rθx)TCx−1Rθx)−1×((Rθx)TCx−1x*+C(0)−1θ¯(0))C(1)=(C(0)−1+(Rθx)TCx−1Rθx)−1.     (10)
 MathType@MTEF@5@5@+=feaafiart1ev1aaatCvAUfKttLearuWrP9MDH5MBPbIqV92AaeXatLxBI9gBaebbnrfifHhDYfgasaacH8akY=wiFfYdH8Gipec8Eeeu0xXdbba9frFj0=OqFfea0dXdd9vqai=hGuQ8kuc9pgc9s8qqaq=dirpe0xb9q8qiLsFr0=vr0=vr0dc8meaabaqaciaacaGaaeqabaqabeGadaaakeaafaqadeWadaaabaacciGaf8hUdeNbaebadaWgaaWcbaGaeiikaGIaeGymaeJaeiykaKcabeaaaOqaaiabg2da9aqaamaabmaabaGaem4qam0aa0baaSqaaiabcIcaOiabicdaWiabcMcaPaqaaiabgkHiTiabigdaXaaakiabgUcaRiabcIcaOiabdkfasnaaDaaaleaacqWF4oqCaeaacqWG4baEaaGccqGGPaqkdaahaaWcbeqaaGqaaiab+rfaubaakiabdoeadnaaDaaaleaacqGF4baEaeaacqGHsislcqaIXaqmaaGccqWGsbGudaqhaaWcbaGae8hUdehabaGaemiEaGhaaaGccaGLOaGaayzkaaWaaWbaaSqabeaacqGHsislcqaIXaqmaaaakeaaaeaaaeaacqGHxdaTdaqadaqaaiabcIcaOiabdkfasnaaDaaaleaacqWF4oqCaeaacqWG4baEaaGccqGGPaqkdaahaaWcbeqaaiab+rfaubaakiabdoeadnaaDaaaleaacqGF4baEaeaacqGHsislcqaIXaqmaaGccqWG4baEcqGGQaGkcqGHRaWkcqWGdbWqdaqhaaWcbaGaeiikaGIaeGimaaJaeiykaKcabaGaeyOeI0IaeGymaedaaOGaf8hUdeNbaebadaWgaaWcbaGaeiikaGIaeGimaaJaeiykaKcabeaaaOGaayjkaiaawMcaaaqaaiabdoeadnaaBaaaleaacqGGOaakcqaIXaqmcqGGPaqkaeqaaaGcbaGaeyypa0dabaWaaeWaaeaacqWGdbWqdaqhaaWcbaGaeiikaGIaeGimaaJaeiykaKcabaGaeyOeI0IaeGymaedaaOGaey4kaSIaeiikaGIaemOuai1aa0baaSqaaiab=H7aXbqaaiabdIha4baakiabcMcaPmaaCaaaleqabaGae4hvaqfaaOGaem4qam0aa0baaSqaaiab+Hha4bqaaiabgkHiTiabigdaXaaakiabdkfasnaaDaaaleaacqWF4oqCaeaacqWG4baEaaaakiaawIcacaGLPaaadaahaaWcbeqaaiabgkHiTiabigdaXaaakiabc6caUaaacaWLjaGaaCzcamaabmaabaGaeGymaeJaeGimaadacaGLOaGaayzkaaaaaa@9084@

These formulae can be obtained by equating the first two terms of (9) to a single quadratic function

(θ−θ¯(0))TC(0)−1(θ−θ¯(0))+(x*−x(θ))TCx−1(x*−x(θ))=(θ−θ¯(1))TC(1)−1(θ−θ¯(1))     (11)
 MathType@MTEF@5@5@+=feaafiart1ev1aaatCvAUfKttLearuWrP9MDH5MBPbIqV92AaeXatLxBI9gBaebbnrfifHhDYfgasaacH8akY=wiFfYdH8Gipec8Eeeu0xXdbba9frFj0=OqFfea0dXdd9vqai=hGuQ8kuc9pgc9s8qqaq=dirpe0xb9q8qiLsFr0=vr0=vr0dc8meaabaqaciaacaGaaeqabaqabeGadaaakeaacqGGOaakiiGacqWF4oqCcqGHsislcuWF4oqCgaqeamaaBaaaleaacqGGOaakcqaIWaamcqGGPaqkaeqaaOGaeiykaKYaaWbaaSqabeaaieaacqGFubavaaGccqWGdbWqdaqhaaWcbaGaeiikaGIaeGimaaJaeiykaKcabaGaeyOeI0IaeGymaedaaOGaeiikaGIae8hUdeNaeyOeI0Iaf8hUdeNbaebadaWgaaWcbaGaeiikaGIaeGimaaJaeiykaKcabeaakiabcMcaPiabgUcaRiabcIcaOiabdIha4jabcQcaQiabgkHiTiabdIha4jabcIcaOiab=H7aXjabcMcaPiabcMcaPmaaCaaaleqabaGae4hvaqfaaOGaem4qam0aa0baaSqaaiab+Hha4bqaaiabgkHiTiabigdaXaaakiabcIcaOiabdIha4jabcQcaQiabgkHiTiabdIha4jabcIcaOiab=H7aXjabcMcaPiabcMcaPiabg2da9iabcIcaOiab=H7aXjabgkHiTiqb=H7aXzaaraWaaSbaaSqaaiabcIcaOiabigdaXiabcMcaPaqabaGccqGGPaqkdaahaaWcbeqaaiab+rfaubaakiabdoeadnaaDaaaleaacqGGOaakcqaIXaqmcqGGPaqkaeaacqGHsislcqaIXaqmaaGccqGGOaakcqWF4oqCcqGHsislcuWF4oqCgaqeamaaBaaaleaacqGGOaakcqaIXaqmcqGGPaqkaeqaaOGaeiykaKIaaCzcaiaaxMaadaqadaqaaiabigdaXiabigdaXaGaayjkaiaawMcaaaaa@7EFE@

and solving for θ¯
 MathType@MTEF@5@5@+=feaafiart1ev1aaatCvAUfKttLearuWrP9MDH5MBPbIqV92AaeXatLxBI9gBaebbnrfifHhDYfgasaacH8akY=wiFfYdH8Gipec8Eeeu0xXdbba9frFj0=OqFfea0dXdd9vqai=hGuQ8kuc9pgc9s8qqaq=dirpe0xb9q8qiLsFr0=vr0=vr0dc8meaabaqaciaacaGaaeqabaqabeGadaaakeaaiiGacuWF4oqCgaqeaaaa@2E81@_(1) _and *C*_(1)_.

### Metabolics-based posterior

In the second step, we consider the metabolic data y* and compute the full posterior (4). The term *p*(y*|*θ*) is hard to compute because *y*(*θ*) depends nonlinearly on *θ*. Therefore, we choose a fixed reference state θ^
 MathType@MTEF@5@5@+=feaafiart1ev1aaatCvAUfKttLearuWrP9MDH5MBPbIqV92AaeXatLxBI9gBaebbnrfifHhDYfgasaacH8akY=wiFfYdH8Gipec8Eeeu0xXdbba9frFj0=OqFfea0dXdd9vqai=hGuQ8kuc9pgc9s8qqaq=dirpe0xb9q8qiLsFr0=vr0=vr0dc8meaabaqaciaacaGaaeqabaqabeGadaaakeaaiiGaliqb=H7aXzaajaaaaa@2E84@ and expand

y(θ)≈y(θ^)+Rθy(θ−θ^).     (12)
 MathType@MTEF@5@5@+=feaafiart1ev1aaatCvAUfKttLearuWrP9MDH5MBPbIqV92AaeXatLxBI9gBaebbnrfifHhDYfgasaacH8akY=wiFfYdH8Gipec8Eeeu0xXdbba9frFj0=OqFfea0dXdd9vqai=hGuQ8kuc9pgc9s8qqaq=dirpe0xb9q8qiLsFr0=vr0=vr0dc8meaabaqaciaacaGaaeqabaqabeGadaaakeaacqWG5bqEcqGGOaakiiGacqWF4oqCcqGGPaqkcqGHijYUcqWG5bqEcqGGOaakcuWF4oqCgaqcaiabcMcaPiabgUcaRiabdkfasnaaDaaaleaacqWF4oqCaeaacqWG5bqEaaGccqGGOaakcqWF4oqCcqGHsislcuWF4oqCgaqcaiabcMcaPiabc6caUiaaxMaacaWLjaWaaeWaaeaacqaIXaqmcqaIYaGmaiaawIcacaGLPaaaaaa@494B@

The matrix Rθy
 MathType@MTEF@5@5@+=feaafiart1ev1aaatCvAUfKttLearuWrP9MDH5MBPbIqV92AaeXatLxBI9gBaebbnrfifHhDYfgasaacH8akY=wiFfYdH8Gipec8Eeeu0xXdbba9frFj0=OqFfea0dXdd9vqai=hGuQ8kuc9pgc9s8qqaq=dirpe0xb9q8qiLsFr0=vr0=vr0dc8meaabaqaciaacaGaaeqabaqabeGadaaakeaacqWGsbGudaqhaaWcbaacciGae8hUdehabaGaemyEaKhaaaaa@313E@ contains the sensitivities Rθmyi
 MathType@MTEF@5@5@+=feaafiart1ev1aaatCvAUfKttLearuWrP9MDH5MBPbIqV92AaeXatLxBI9gBaebbnrfifHhDYfgasaacH8akY=wiFfYdH8Gipec8Eeeu0xXdbba9frFj0=OqFfea0dXdd9vqai=hGuQ8kuc9pgc9s8qqaq=dirpe0xb9q8qiLsFr0=vr0=vr0dc8meaabaqaciaacaGaaeqabaqabeGadaaakeaacqWGsbGudaqhaaWcbaacciGae8hUde3aaSbaaWqaaiabd2gaTbqabaaaleaacqWG5bqEdaWgaaadbaGaemyAaKgabeaaaaaaaa@3461@ = ∂*y*_*i*_/∂*θ*_*m*_. The posterior for this linearised model is a multivariate Gaussian distribution N
 MathType@MTEF@5@5@+=feaafiart1ev1aaatCvAUfKttLearuWrP9MDH5MBPbIqV92AaeXatLxBI9gBamrtHrhAL1wy0L2yHvtyaeHbnfgDOvwBHrxAJfwnaebbnrfifHhDYfgasaacH8akY=wiFfYdH8Gipec8Eeeu0xXdbba9frFj0=OqFfea0dXdd9vqai=hGuQ8kuc9pgc9s8qqaq=dirpe0xb9q8qiLsFr0=vr0=vr0dc8meaabaqaciaacaGaaeqabaWaaeGaeaaakeaaimaacqWFneVtaaa@383B@(θ¯
 MathType@MTEF@5@5@+=feaafiart1ev1aaatCvAUfKttLearuWrP9MDH5MBPbIqV92AaeXatLxBI9gBaebbnrfifHhDYfgasaacH8akY=wiFfYdH8Gipec8Eeeu0xXdbba9frFj0=OqFfea0dXdd9vqai=hGuQ8kuc9pgc9s8qqaq=dirpe0xb9q8qiLsFr0=vr0=vr0dc8meaabaqaciaacaGaaeqabaqabeGadaaakeaaiiGacuWF4oqCgaqeaaaa@2E81@_(2)_, *C*_(2)_) with mean and covariance matrix

θ¯(2)=θ^+(C(1)−1+(Rθy)TCy−1Rθy)−1×((Rθy)TCy−1(y*−y(θ^))+C(1)−1(θ¯(1)−θ^))C(2)=(C(1)−1+(Rθy)TCy−1Rθy)−1.     (13)
 MathType@MTEF@5@5@+=feaafiart1ev1aaatCvAUfKttLearuWrP9MDH5MBPbIqV92AaeXatLxBI9gBaebbnrfifHhDYfgasaacH8akY=wiFfYdH8Gipec8Eeeu0xXdbba9frFj0=OqFfea0dXdd9vqai=hGuQ8kuc9pgc9s8qqaq=dirpe0xb9q8qiLsFr0=vr0=vr0dc8meaabaqaciaacaGaaeqabaqabeGadaaakeaafaqadeWadaaabaacciGaf8hUdeNbaebadaWgaaWcbaGaeiikaGIaeGOmaiJaeiykaKcabeaaaOqaaiabg2da9aqaaiqb=H7aXzaajaaccaGae43kaSYaaeWaaeaacqWGdbWqdaqhaaWcbaGaeiikaGIaeGymaeJaeiykaKcabaGaeyOeI0IaeGymaedaaOGaey4kaSIaeiikaGIaemOuai1aa0baaSqaaiab=H7aXbqaaiabdMha5baakiabcMcaPmaaCaaaleqabaacbaGae0hvaqfaaOGaem4qam0aa0baaSqaaiab9Lha5bqaaiabgkHiTiabigdaXaaakiabdkfasnaaDaaaleaacqWF4oqCaeaacqWG5bqEaaaakiaawIcacaGLPaaadaahaaWcbeqaaiabgkHiTiabigdaXaaaaOqaaaqaaaqaaiabgEna0oaabmaabaGaeiikaGIaemOuai1aa0baaSqaaiab=H7aXbqaaiabdMha5baakiabcMcaPmaaCaaaleqabaGae0hvaqfaaOGaem4qam0aa0baaSqaaiab9Lha5bqaaiabgkHiTiabigdaXaaakiabcIcaOiabdMha5jabcQcaQiabgkHiTiabdMha5jabcIcaOiqb=H7aXzaajaGaeiykaKIaeiykaKIaey4kaSIaem4qam0aa0baaSqaaiabcIcaOiabigdaXiabcMcaPaqaaiabgkHiTiabigdaXaaakiab9HcaOiqb=H7aXzaaraWaaSbaaSqaaiabcIcaOiabigdaXiabcMcaPaqabaGccqGHsislcuWF4oqCgaqcaiab9LcaPaGaayjkaiaawMcaaaqaaiabdoeadnaaBaaaleaacqGGOaakcqaIYaGmcqGGPaqkaeqaaaGcbaGaeyypa0dabaWaaeWaaeaacqWGdbWqdaqhaaWcbaGaeiikaGIaeGymaeJaeiykaKcabaGaeyOeI0IaeGymaedaaOGaey4kaSIaeiikaGIaemOuai1aa0baaSqaaiab=H7aXbqaaiabdMha5baakiabcMcaPmaaCaaaleqabaGae0hvaqfaaOGaem4qam0aa0baaSqaaiab9Lha5bqaaiabgkHiTiabigdaXaaakiabdkfasnaaDaaaleaacqWF4oqCaeaacqWG5bqEaaaakiaawIcacaGLPaaadaahaaWcbeqaaiabgkHiTiabigdaXaaakiabc6caUaaacaWLjaGaaCzcamaabmaabaGaeGymaeJaeG4mamdacaGLOaGaayzkaaaaaa@9F2A@

The formula has a similar form as (10): in fact, we use the first posterior as a new prior for the second step. We use eqn. (13) to approximate the posterior of the nonlinear model. For the expansion point θ^
 MathType@MTEF@5@5@+=feaafiart1ev1aaatCvAUfKttLearuWrP9MDH5MBPbIqV92AaeXatLxBI9gBaebbnrfifHhDYfgasaacH8akY=wiFfYdH8Gipec8Eeeu0xXdbba9frFj0=OqFfea0dXdd9vqai=hGuQ8kuc9pgc9s8qqaq=dirpe0xb9q8qiLsFr0=vr0=vr0dc8meaabaqaciaacaGaaeqabaqabeGadaaakeaaiiGaliqb=H7aXzaajaaaaa@2E84@, we choose the centre of the posterior; therefore, we need to find a self-consistent solution in which the expansion point and the posterior mode match [See Additional file 1].

As an initial guess, we choose model parameters that are guaranteed to yield a steady state: we set all kinetic parameters and all concentrations equal to one; in this state, all reaction velocities vanish and we obtain a thermal equilibrium. We then compute the posterior that results from the linearised model, move our expansion point towards the parameter set θ¯
 MathType@MTEF@5@5@+=feaafiart1ev1aaatCvAUfKttLearuWrP9MDH5MBPbIqV92AaeXatLxBI9gBaebbnrfifHhDYfgasaacH8akY=wiFfYdH8Gipec8Eeeu0xXdbba9frFj0=OqFfea0dXdd9vqai=hGuQ8kuc9pgc9s8qqaq=dirpe0xb9q8qiLsFr0=vr0=vr0dc8meaabaqaciaacaGaaeqabaqabeGadaaakeaaiiGacuWF4oqCgaqeaaaa@2E81@_(2)_, and iterate the whole procedure until convergence. The computational complexity of the algorithm depends on the convergence of the iteration scheme, which varies from model to model. We found that the first estimation step is computationally cheap compared to the repeated computation of steady states that are necessary for the second posterior.

## Test case

### Threonine model

**Figure 3:**
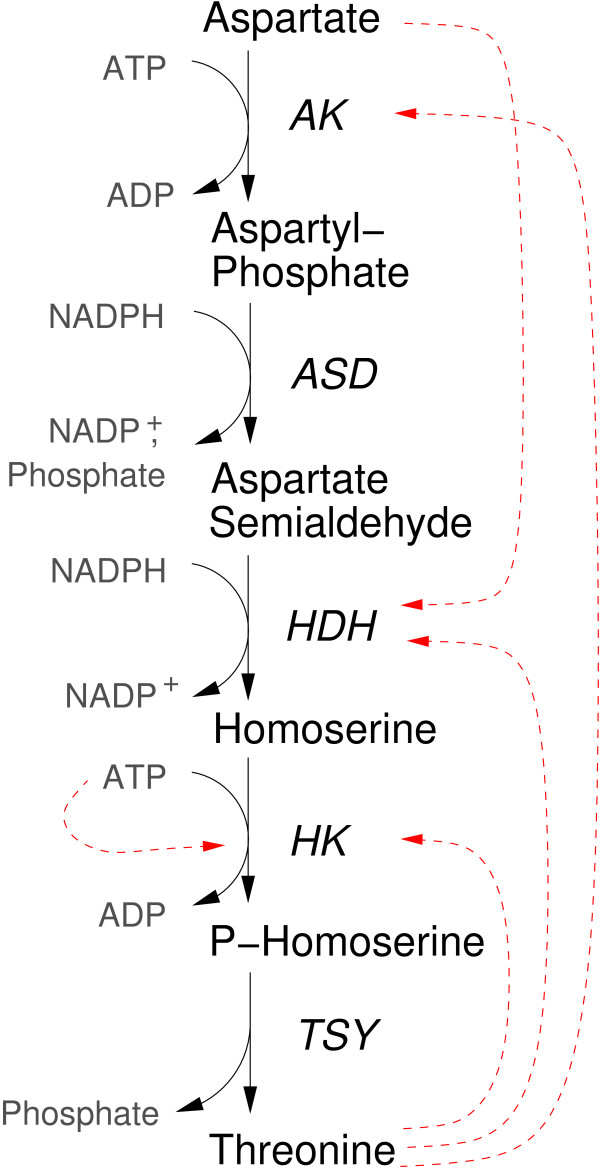
**Threonine biosynthesis pathway**. The chemical reactions are catalysed by aspartate kinase (AK), aspartate semialdehyde dehydrogenase (ASD), homoserine dehydrogenase (HDH), homoserine kinase (HK), and threonine synthase (TSY). Metabolites with fixed and variable concentrations are shown as grey and white boxes, respectively. Solid arrows denote production and consumption of metabolites, red dashed arrows denote enzyme inhibition.

The threonine biosynthesis pathway converts aspartate into threonine with the consumption of ATP and NADPH (Figure 3). A detailed kinetic model of the pathway has been presented by Chassagnole et al. [33]. To test our method, we simulated the threonine pathway with a (hypothetical) convenience kinetics and generated noisy artificial data. We regard all cofactors and the end points of the pathway as buffered and treat their concentrations as fixed. The concentrations of the four intermediates aspartyl-phosphate, aspartate semialdehyde, homoserine, and P-homoserine are the dynamical variables. The kinetic parameters were chosen such as to mimic the model of Chassagnole et al. [33].

The model parameters were reestimated from the artificial data, comprising noisy kinetic parameters, metabolite and enzyme concentrations, and metabolic fluxes. As prior distributions, we used log-normal distributions fitted to the empirical parameter distributions shown in table 1. Details of the model and the computation are described [See Additional file 1].

### Estimation results

**Figure 4:**
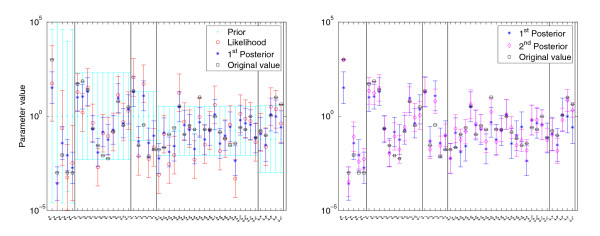
**Posterior distributions in the threonine model**. Left: prior and kinetics-based posterior in the threonine model. All system kinetic parameters (energy constants kiG
 MathType@MTEF@5@5@+=feaafiart1ev1aaatCvAUfKttLearuWrP9MDH5MBPbIqV92AaeXatLxBI9gBaebbnrfifHhDYfgasaacH8akY=wiFfYdH8Gipec8Eeeu0xXdbba9frFj0=OqFfea0dXdd9vqai=hGuQ8kuc9pgc9s8qqaq=dirpe0xb9q8qiLsFr0=vr0=vr0dc8meaabaqaciaacaGaaeqabaqabeGadaaakeaacqWGRbWAdaqhaaWcbaGaemyAaKgabaacbaGae83raCeaaaaa@30AF@, velocity constants klV
 MathType@MTEF@5@5@+=feaafiart1ev1aaatCvAUfKttLearuWrP9MDH5MBPbIqV92AaeXatLxBI9gBaebbnrfifHhDYfgasaacH8akY=wiFfYdH8Gipec8Eeeu0xXdbba9frFj0=OqFfea0dXdd9vqai=hGuQ8kuc9pgc9s8qqaq=dirpe0xb9q8qiLsFr0=vr0=vr0dc8meaabaqaciaacaGaaeqabaqabeGadaaakeaacqWGRbWAdaqhaaWcbaGaemiBaWgabaacbaGae8Nvayfaaaaa@30D3@, *k*^M ^and *k*^I ^values) and the equilibrium constants kieq
 MathType@MTEF@5@5@+=feaafiart1ev1aaatCvAUfKttLearuWrP9MDH5MBPbIqV92AaeXatLxBI9gBaebbnrfifHhDYfgasaacH8akY=wiFfYdH8Gipec8Eeeu0xXdbba9frFj0=OqFfea0dXdd9vqai=hGuQ8kuc9pgc9s8qqaq=dirpe0xb9q8qiLsFr0=vr0=vr0dc8meaabaqaciaacaGaaeqabaqabeGadaaakeaacqWGRbWAdaqhaaWcbaGaemyAaKgabaacbaGae8xzauMae8xCaehaaaaa@3252@ are listed on the abscissa. Black □: parameter values from the original model. Bars of different colours represent the marginal distributions (mean and standard deviation), corresponding to the arrows in the left diagram. Light blue ●: prior distribution of the logarithmic parameters. Red ○: likelihood function representing artificial experimental values with error bars. Dark blue *: kinetics-based posterior distribution. Right: true values (black □) and first, kinetics-based posterior (blue bars, *). Second, metabolics-based posterior (purple bars, ◇) computed from artificial data. The marginal distributions of kinetics-based and metabolics-based posteriors look quite similar.

**Figure 5:**
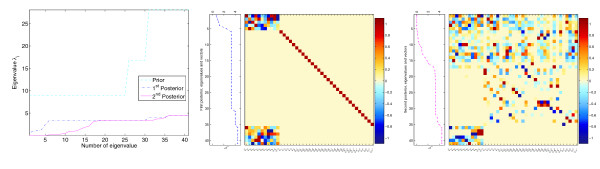
**Joint distribution in the threonine model**. Left: eigenvalues of the covariance matrices *C*_(0) _(light blue - - for prior), *C*_(1) _(dark blue -.-, first posterior), *C*_(2) _(purple —, second posterior). The width of the parameter distribution decreases in both estimation steps. Some eigenvalues become very small in the second posterior; they represent well-defined parameter combinations. Centre: eigenvectors for the first posterior. Each row of the matrix corresponds to an eigenvector (normalised to a maximal value of 1 for the elements). The corresponding eigenvalues are shown in the box on the left. The distribution of energy constants is well-defined in some directions (eigenvectors on top, with low eigenvalues) and uncertain in other directions (bottom, high eigenvalues). The *k*^M ^and *k*^I ^values are uncorrelated (described by individual eigenvectors). Right: the eigenvectors of the second posterior fall into three groups: (i) eigenvectors for well-defined directions, coupling all sorts of parameters (top), (ii) less well-defined combinations of *k*^M ^and *k*^I ^values (centre), and (iii) poorly defined combinations of energy constants (bottom).

The resulting parameter distributions are shown in Figure (4). As expected, integration of data improves the accuracy of the predictions: the resulting probability densities, evaluated at the original parameter set *θ*^kin^, increase in both steps: *p*(*θ*^kin^) <*p*(*θ*^kin^|x*) <*p*(*θ*^kin^|y*, x*). Figure 4, left, shows the prior and the kinetics-based posterior for the system parameters and for the equilibrium constants. The first estimation step narrows down the marginal parameter distributions compared to the prior distribution. Incorporation of the metabolic data further improves the accuracy, as shown in Figure 4, right. The marginal distributions change only slightly, but the correlations between the parameters become stronger. The eigenvalues of the covariance matrices (Figure 5) show that in certain directions in parameter space, the joint distribution becomes very narrow. In other directions, the distribution remains broad: the six largest eigenvalues correspond to the linear combinations of energy constants kiG
 MathType@MTEF@5@5@+=feaafiart1ev1aaatCvAUfKttLearuWrP9MDH5MBPbIqV92AaeXatLxBI9gBaebbnrfifHhDYfgasaacH8akY=wiFfYdH8Gipec8Eeeu0xXdbba9frFj0=OqFfea0dXdd9vqai=hGuQ8kuc9pgc9s8qqaq=dirpe0xb9q8qiLsFr0=vr0=vr0dc8meaabaqaciaacaGaaeqabaqabeGadaaakeaacqWGRbWAdaqhaaWcbaGaemyAaKgabaacbaGae83raCeaaaaa@30AF@ that leave all equilibrium constants unchanged. These combinations do not affect the metabolic behaviour, so they are not identifiable from metabolic data.

### Model predictions

**Figure 6:**
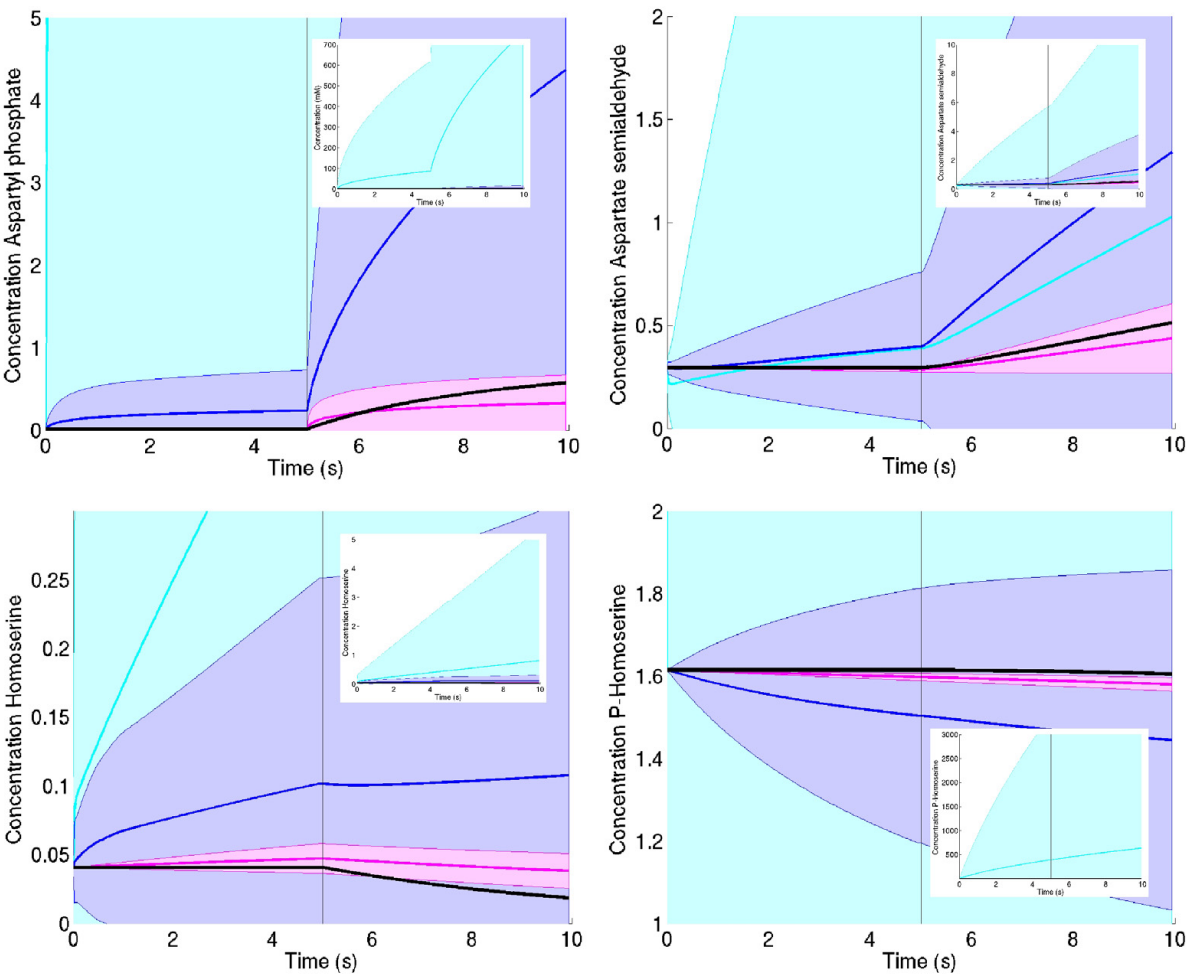
**Simulation results for threonine model**. The refined parameter distributions lead to better predictions of the dynamic behaviour. Top left: simulated time series for aspartyl-phosphate. The curve from the true model is shown by black squares. After five minutes, the substrate aspartate is shifted to a higher concentration, leading to an increase of aspartyl-phosphate. Each parameter ensemble creates a distribution of simulation results: areas represent the standard deviations, the colours represent prior (light blue), kinetics-based posterior (dark blue) and metabolics-based posterior (purple). Inset: other scaling to show the relative spread of prior and first posterior. Other diagrams: time series for the remaining metabolites aspartate semialdehyde (top right), homoserine (bottom left), and p-homoserine (bottom right).

Do better parameter estimates also improve predictions about the dynamical behaviour? As a test, we simulated the threonine model with parameter sets sampled from the prior, the first posterior, and the second posterior. To assess how the time courses are distributed, we simulated the system 100 times with random parameters drawn from the respective distribution. Figure 6 shows the spread of concentration time courses that resulted from the sampled models. In the first half of the time series, the steady-state concentrations of the original model were used as initial conditions. After the first half, the aspartate concentration was increased by a factor of 50.

We found that the accuracy of the predictions increased considerably between the kinetics-based and the metabolics-based posterior. Hence, the fit to metabolic data adds important information to the parameter ensemble; this information is contained in the parameter correlations rather than in the marginal distributions.

## Discussion

We proposed a method to construct kinetic models from biochemical networks: all reactions are modelled by convenience kinetics, and the parameters are characterised by a posterior distribution. We approximate the posterior by a multivariate log-normal distribution, or in other words, by a Gaussian distribution for the logarithmic parameters.

The convenience kinetics is a simple and biologically sensible choice when the reaction mechanisms are unknown. Other kinetic laws can be used just as well if the kinetic parameters can be expressed by thermodynamically independent parameters that obey an equation of form (8). This holds for many kinetic laws including mass-action kinetics and laws of the Michaelis-Menten type. Parameters such as activation and inhibition constants, which do not affect the chemical equilibrium, can be chosen independently. The posterior distribution represents a compromise between the typical ranges of model parameters and a fit to specific experimental data. Data sources with small error bars will have the greatest impact in the estimation. If the model is fitted to sparse and unreliable data, the parameters will be poorly determined, and the remaining uncertainty can be read from the parameter distribution. If new data become available, the model parameters can be easily reestimated, using the old posterior distribution as a prior for the next parameter fit. For simplicity, we assumed here that metabolic data are given in absolute numbers. If only relative data are available, appropriate scaling factors have to be estimated along with the other model parameters. Instead of steady state data, metabolic time series may also be used in the estimation – in this case, the time-dependent protein concentrations have to be interpolated, and time-dependent response coefficients [36] are used in the calculation. It is of course also possible to use the goal function (9) with other parameter estimation algorithms.

The use of logarithmic parameters enabled us to describe relations between the parameters by linear equations and to use Gaussian distributions. As the parameter vector *θ *contains logarithmic values, our Gaussian prior actually represents a log-normal distribution of the kinetic parameters. The same holds for the likelihood given the kinetic data x* in eqn. (6). In contrast to that, the metabolic data y* in (7) are used in their non-logarithmic form. Why? Metabolic fluxes can become negative, and then the log-transformation is not possible. This problem can be avoided by splitting the fluxes into forward and backward components [15], and then our estimation method can also be applied to metabolic data in logarithmic form. After all, the choice between use of logarithmic and non-logarithmic data reflects our assumption about the noise term: with non-logarithmic data, it represents additive Gaussian noise. If logarithmic data are used, the same model represents multiplicative log-normal noise in the original data. 

Our approach is limited by the two approximations made: (i) the true reaction kinetics are replaced by convenience kinetics; (ii) to compute the posterior, the model is linearised around a posterior mode. Nevertheless, automatic parameter estimation can provide reasonable first guesses and plausible ranges of model parameters. Kinetic parameters obtained from the integration of many literature values and incorporation of thermodynamic constraints are probably more reliable than the single literature values.

## Conclusion

To simulate a biochemical system, the network structure, the kinetic laws, and the kinetic parameters must be determined. Usually, this process involves literature studies and several iteration cycles of experiments, parameter fitting, and model selection. We have presented a method to guess model parameters by integrating existing kinetic, metabolic, and proteomic data. The parameters are described by a posterior parameter distribution that summarises the information extracted from the experimental data. A model with the mean logarithmic parameters matches the known experimental data as closely as possible and gives an impression of the dynamic behaviour. The covariance matrix describes the remaining uncertainties and the correlations between the parameters; by sampling from the parameter distribution, we can simulate more and more model instances and explore their behaviour. If the parameter distribution is narrow, then metabolic concentrations and fluxes deviate little from the typical behaviour, and their distribution can be approximated by analytical calculation [15].

The estimation procedure can be split into two separate steps: first, the kinetic parameters in the model are fitted to kinetic and thermodynamic data; second, the parameters are improved by fitting them to metabolic steady states. In our computational example, incorporating the metabolic data increased the accuracy of prediction; the improvement seems to be caused by the parameter correlations rather than by narrower marginal distributions of the individual parameters.

The use of thermodynamically independent parameters ensures that all models respect the second law of thermodynamics. We presented an algorithm to approximate the posterior by a multivariate Gaussian distribution. The result is a mathematical model with uncertain parameters; it can be used to compute probabilities for the system behaviour by sampling, simulation, and analysis of model instances.  Model ensembles as presented here can help to assess the dynamic effects of the model structure, bridging the gap between pathway analysis, enzyme kinetic databases, and kinetic modelling.

## Methods

### Empirical distributions of kinetic parameters

We obtained prior distributions for different types of parameters from statistics over experimental data  [18][19,20,23,24]. The results are shown in table 1.

1. Experimental values for turnover rates, substrate, product, and inhibition constants were taken from the Brenda database [18]. The database contains multiple values for some of the parameters; we counted them separately.

2. To obtain energy constants, we used Gibbs free energies of formation predicted from the molecule structures, using the group contribution method [23]: values for CoA-complexes were neglected in the statistics, and the values for the remaining compounds were -590 ± 447 J/mol. We computed the values of the energy constants kiG=eG(0)/(RT)
 MathType@MTEF@5@5@+=feaafiart1ev1aaatCvAUfKttLearuWrP9MDH5MBPbIqV92AaeXatLxBI9gBaebbnrfifHhDYfgasaacH8akY=wiFfYdH8Gipec8Eeeu0xXdbba9frFj0=OqFfea0dXdd9vqai=hGuQ8kuc9pgc9s8qqaq=dirpe0xb9q8qiLsFr0=vr0=vr0dc8meaabaqaciaacaGaaeqabaqabeGadaaakeaacqWGRbWAdaqhaaWcbaGaemyAaKgabaacbaGae83raCeaaOGaeyypa0Jae8xzau2aaWbaaSqabeaacqWGhbWrdaahaaadbeqaaiabcIcaOiabicdaWiabcMcaPaaaliabc+caViabcIcaOiabdkfasjabdsfaujabcMcaPaaaaaa@3C21@ using the gas constant *R *≈ 8.314 J/(mol K) and a temperature of 300 K (approximately 25°C), thus *RT *≈ 2.490 kJ/mol.

3. Enzyme concentrations were roughly guessed from protein molecule numbers in the yeast *S. cerevisiae*, measured in a GFP assay [20]. To convert molecule numbers into concentrations, we assumed a spherical cell of radius 6 *μ*m. The protein concentration reads *c *= *N*_molecules_/(*N*_*A*_*V*_cell_) M, with Avogadro's constant *N*_*A *_= 6.022 · 10^23 ^and the cell volume measured in litres.

4. The concentrations of 49 metabolites were taken from a literature survey [24]. Concentrations measured in different species were averaged as described [37].

5. Equilibrium constants were taken from the NIST data base [19]. The physical units mM, 1, and mM depend on the reaction stoichiometry, but we describe all numerical values by a single distribution. This is justified as long as we are only interested in the reaction Gibbs free energies that correspond to the equilibrium constants. To avoid bias due to the arbitrary choice of the standard reaction directions, we counted each reaction in both forward and backward directions. Hence, the mean value has no meaningful interpretation.

We found that the distributions of computed Gibbs free energies of formation did not agree with the distribution of equilibrium constants. Thus, for the energy constants ln kiG
 MathType@MTEF@5@5@+=feaafiart1ev1aaatCvAUfKttLearuWrP9MDH5MBPbIqV92AaeXatLxBI9gBaebbnrfifHhDYfgasaacH8akY=wiFfYdH8Gipec8Eeeu0xXdbba9frFj0=OqFfea0dXdd9vqai=hGuQ8kuc9pgc9s8qqaq=dirpe0xb9q8qiLsFr0=vr0=vr0dc8meaabaqaciaacaGaaeqabaqabeGadaaakeaacqWGRbWAdaqhaaWcbaGaemyAaKgabaacbaGae83raCeaaaaa@30AF@ = *G*_*i*_/(*RT*) in the threonine model, we chose a different prior, with a mean value of zero and a standard deviation of In 200 ≈ 5.3.

## Competing interests

The authors declare that they have no competing interests.

## Authors' contributions

W. L. conceived the method, carried out calculations, and wrote the manuscript. E. K. revised the manuscript. Both authors read and approved the final manuscript.

## Supplementary Material

Additional file 1 The supplementary file contains a list of the mathematical symbols used, a description of the threonine model, and an algorithm for approximating the posterior parameter distribution. Click here for file
