# Supplementary material for: Bringing metabolic networks to life: integration of kinetic, metabolic, and proteomic data
Source: Theor Biol Med Model. 2006 Dec 15;3:42. doi: 10.1186/1742-4682-3-42 (PMC1781439; doi:10.1186/1742-4682-3-42)
Supplement: Additional file 1 — The supplementary file contains a list of the mathematical symbols used, a description of the threonine model, and an algorithm for approximating the posterior parameter distribution. [file 1742-4682-3-42-S1.pdf]

Bringing metabolic networks to life: integration of kinetic, metabolic, and proteomic data

Supplementary material

Wolfram Liebermeister[1]*, Edda Klipp[1]

[1]Computational Systems Biology, Max Planck Institute for Molecular Genetics,
Ihnestraße 63-73, 14195 Berlin, Germany

Email: Wolfram Liebermeister - lieberme@molgen.mpg.de; Edda Klipp - klipp@molgen.mpg.de;

*Corresponding author

Table S1 - List of mathematical symbols

Symbol	Name
$N = (n_{il})$	stoichiometric matrix
$W = (w_{li})$	regulation matrix
$s = (s_i)$	vector of stationary metabolite concentrations
$j = (j_l)$	vector of stationary fluxes
$v_l(\cdot)$	kinetic law of reaction l
k_i^{G}	energy constant for metabolite i
k_l^{V}	velocity constant for reaction l
k_{li}^{M}	reactant constant for reaction l and metabolite i
k_{li}^{A}	activation constant for reaction l and metabolite i
k_{li}^{I}	inhibition constant for reaction l and metabolite i
$k_{\pm l}^{\mathrm{cat}}$	maximal turnover rates (forward and backward) for reaction l
E_l	enzyme concentration for reaction l
$v_{\pm l}^{\mathrm{max}}$	maximal velocities (forward and backward) for reaction l
θ	vector of all model parameters (logarithmic)
x	vector of all measured kinetic parameters (logarithmic)
y	vector of all measured metabolic data (non-logarithmic)
R_θ^x	sensitivity matrix between system parameters and kinetic data
R_θ^y	sensitivity matrix between system parameters and metabolic data
$\bar{\theta}_{(0)}, C_{(0)}$	mean vector and covariance matrix for prior of θ
$\bar{\theta}_{(1)}, C_{(1)}$	mean vector and covariance matrix for first posterior of θ
$\bar{\theta}_{(2)}, C_{(2)}$	mean vector and covariance matrix for second posterior of θ
$\hat{\theta}$	expansion point for θ
$\hat{s}^{(m)}$	vector of initial concentrations for computing the steady state m

The threonine model

Network structure

We downloaded the model in SBML format [1] from the model database JWS online [2]. The chemical reactions are catalysed by aspartate kinase (AK), aspartate semialdehyde dehydrogenase (ASD), homoserine dehydrogenase (HDH), homoserine kinase (HK), threonine synthase (TSY). In our model, the aspartate kinase reaction is represented by a single enzyme, AKI, while the isoenzyme AKIII was neglected. Protons and water also participate in the pathway but are not explicitly described by the model. The stoichiometric matrix N of the network reads

$$N = \left(\begin{array}{ccccc} \text{AK} & \text{ASD} & \text{HDH} & \text{HK} & \text{TSY} \\ \hline 1 & -1 & \cdot & \cdot & \cdot \\ \cdot & 1 & -1 & \cdot & \cdot \\ \cdot & \cdot & 1 & -1 & \cdot \\ \cdot & \cdot & \cdot & 1 & -1 \\ \cdot & 1 & \cdot & \cdot & 1 \\ \cdot & \cdot & \cdot & \cdot & 1 \\ -1 & \cdot & \cdot & \cdot & \cdot \\ \cdot & 1 & 1 & \cdot & \cdot \\ \cdot & -1 & -1 & \cdot & \cdot \\ 1 & \cdot & \cdot & 1 & \cdot \\ -1 & \cdot & \cdot & -1 & \cdot \end{array}\right) \begin{array}{l} \\ \text{4-phospho-l-aspartate} \\ \text{l-aspartate 4-semialdehyde} \\ \text{l-homoserine} \\ \text{o-phospho-l-homoserine} \\ \text{orthophosphate} \\ \text{l-threonine} \\ \text{l-aspartate} \\ \text{NADP+} \\ \text{NADPH} \\ \text{ADP} \\ \text{ATP} \end{array} \tag{1}$$

In steady state, production and consumption of the internal compounds (aspartyl-phosphate, aspartate semialdehyde, homoserine, and P-homoserine) are balanced, which implies that all five reactions have the same velocity. The transposed regulation matrix W^{T} reads

$$W = \left(\begin{array}{ccccc} \text{AK} & \text{ASD} & \text{HDH} & \text{HK} & \text{TSY} \\ \hline \cdot & \cdot & \cdot & \cdot & \cdot \\ \cdot & \cdot & \cdot & \cdot & \cdot \\ \cdot & \cdot & \cdot & \cdot & \cdot \\ \cdot & \cdot & \cdot & \cdot & \cdot \\ \cdot & \cdot & \cdot & \cdot & \cdot \\ -1 & \cdot & -1 & -1 & \cdot \\ \cdot & \cdot & -1 & \cdot & \cdot \\ \cdot & \cdot & \cdot & \cdot & \cdot \\ \cdot & \cdot & \cdot & \cdot & \cdot \\ \cdot & \cdot & \cdot & \cdot & \cdot \\ \cdot & \cdot & \cdot & -1 & \cdot \end{array}\right) \begin{array}{l} \\ \text{4-phospho-l-aspartate} \\ \text{l-aspartate 4-semialdehyde} \\ \text{l-homoserine} \\ \text{o-phospho-l-homoserine} \\ \text{orthophosphate} \\ \text{l-threonine} \\ \text{l-aspartate} \\ \text{NADP+} \\ \text{NADPH} \\ \text{ADP} \\ \text{ATP} \end{array} \tag{2}$$

Note that ATP acts both as a substrate and as an inhibitor of the homoserine kinase (HK) reaction.

Hypothetical kinetics

For testing our method, we created a model of the threonine pathway with a hypothetical convenience kinetics. The original parameters were chosen similar to the experimentally determined kinetics in Chassagnole et al. [3]. The values are listed in table S2. For the calculations, the remaining k^{M}_{li}, k^{A}_{li}, and k^{I}_{li} values that do not correspond to a stoichiometric or regulatory connection were set to the arbitrary value of 1, that is, a logarithmic value of 0. Artificial experimental values for the kinetic parameters were generated by adding Gaussian noise (of width $\ln 10$) to the original logarithmic enzyme parameters.

To create artificial metabolic data, we randomly chose concentrations for the fixed metabolites and the enzymes by multiplying the standard values with independent log-normal random numbers. We computed steady states for five such random assignments and repeated this ten times. The resulting steady state quantities (concentrations, fluxes, enzyme concentrations) were used as artificial data. As a prior for the logarithmic system parameters, we chose an uncorrelated Gaussian distribution with mean values and standard deviations as given in table 1 (see also methods section).

Time series for the original model and the sampled models were computed as follows: we chose the steady-state concentrations of the original model as initial conditions for all simulations. For the second half of the time series, the concentrations of the external metabolite aspartate was increased by a factor of 50.

Table S2 - Kinetic parameters of the hypothetical threonine pathway model

$G^{(0)}_{\mathrm{P-l-aspartate}}$	3.94	kJ/mol/RT
$G^{(0)}_{\mathrm{aspartatesemiald.}}$	4.28	kJ/mol/RT
$G^{(0)}_{\mathrm{homoserine}}$	3.21	kJ/mol/RT
$G^{(0)}_{\mathrm{P-homoserine}}$	-1.47	kJ/mol/RT
$G^{(0)}_{\mathrm{orthophosphate}}$	-3.57	kJ/mol/RT
$G^{(0)}_{\mathrm{threonine}}$	-4.80	kJ/mol/RT
$G^{(0)}_{\mathrm{aspartate}}$	-5.17	kJ/mol/RT
$G^{(0)}_{\mathrm{NADP+}}$	-1.83	kJ/mol/RT
$G^{(0)}_{\mathrm{NADPH}}$	1.83	kJ/mol/RT
$G^{(0)}_{\mathrm{ADP}}$	-1.10	kJ/mol/RT
$G^{(0)}_{\mathrm{ATP}}$	1.10	kJ/mol/RT

$k^{\mathrm{M}}_{1,1}$	0.017	mM
$k^{\mathrm{M}}_{2,1}$	0.022	mM
$k^{\mathrm{M}}_{2,2}$	0.11	mM
$k^{\mathrm{M}}_{3,2}$	0.24	mM
$k^{\mathrm{M}}_{3,3}$	3.39	mM
$k^{\mathrm{M}}_{5,3}$	0.11	mM
$k^{\mathrm{M}}_{4,4}$	0.31	mM
$k^{\mathrm{M}}_{5,4}$	0.2	mM
$k^{\mathrm{M}}_{2,5}$	10.2	mM
$k^{\mathrm{M}}_{4,5}$	0.2	mM
$k^{\mathrm{M}}_{4,6}$	0.2	mM
$k^{\mathrm{M}}_{1,7}$	0.97	mM
$k^{\mathrm{M}}_{2,8}$	0.144	mM
$k^{\mathrm{M}}_{3,8}$	0.067	mM
$k^{\mathrm{M}}_{2,9}$	0.029	mM
$k^{\mathrm{M}}_{3,9}$	0.037	mM
$k^{\mathrm{M}}_{1,10}$	0.25	mM
$k^{\mathrm{M}}_{5,10}$	0.2	mM
$k^{\mathrm{M}}_{1,11}$	0.98	mM
$k^{\mathrm{M}}_{5,11}$	0.072	mM

$k^{\mathrm{V}}_{\mathrm{AKA}}$	21.81	s^{-1}
$k^{\mathrm{V}}_{\mathrm{ASD}}$	0.02	s^{-1}
$k^{\mathrm{V}}_{\mathrm{HDH}}$	0.34	s^{-1}
$k^{\mathrm{V}}_{\mathrm{HK}}$	0.0064	s^{-1}
$k^{\mathrm{V}}_{\mathrm{TSY}}$	0.017	s^{-1}
$k^{\mathrm{I}}_{1,6}$	0.167	mM
$k^{\mathrm{I}}_{3,6}$	0.097	mM
$k^{\mathrm{I}}_{5,6}$	1.09	mM
$k^{\mathrm{I}}_{3,7}$	10	mM
$k^{\mathrm{I}}_{5,11}$	4.35	mM
$c_{\mathrm{orthophosphate}}$	5	mM
$c_{\mathrm{threonine}}$	3.49	mM
$c_{\mathrm{aspartate}}$	1.34	mM
$c_{\mathrm{NADP+}}$	0.63	mM
c_{NADPH}	0.56	mM
c_{ADP}	0.17	mM
c_{ATP}	1.31	mM

The numerical subscripts refer to the numbering of substances and reactions in the transposed stoichiometric matrix (see above).

An algorithm for computing the posterior distribution

First step: integration of kinetic parameters

In the first estimation step, only the system parameters θ^{kin} have to be fitted. We indicate the respective quantities by a superscript (like in $\bar{\theta}^{\mathrm{kin}}_{(0)}$). The algorithm reads

1. Parse the model (for instance, in SBML format [1]) and construct the network matrices N and W.
2. Parse the available experimental data for kinetic parameters and collect them in a vector x^{*}. Parse or assume experimental error levels σ_{x} and construct the corresponding covariance matrix C_{x}.
3. Construct the sensitivity matrix $R^{x}_{\theta^{\mathrm{kin}}}$ from N and W (see [4]).
4. Choose a Gaussian prior distribution for the system parameters. Construct the vector $\bar{\theta}^{\mathrm{kin}}_{(0)}$ and the matrix $C^{\mathrm{kin}}_{(0)}$ from mean values and standard deviations of parameter types (for numerical values, see table 1).
5. Compute $\bar{\theta}^{\mathrm{kin}}_{(1)}$ and $C^{\mathrm{kin}}_{(1)}$ for the kinetics-based posterior, using eqn. (10) and the matrix $R^{x}_{\theta^{\mathrm{kin}}}$ instead of R^{x}_{θ}.

To avoid costly matrix inversions, we compute mathematical expressions of the form $A^{-1}x$ (with a sparse matrix A and a vector x) by Gaussian elimination ("left matrix divide" in MATLAB).

Second step: integration of metabolic data

The algorithm for the second step, the metabolics-based parameter estimation, is more complicated. Here we assume that metabolic quantities have been measured for a couple of steady states (indicated by m). Each steady state results from certain concentrations of enzymes and fixed metabolites, which are included in the model as model parameters θ^{met} (logarithmic values). Moreover, for each steady state, we have to guess initial concentrations for all metabolites. In the end, the conserved moieties of the initial concentrations must be compatible with the conserved moieties of the experimental concentrations. In the algorithm, we start with parameters and initial concentrations that correspond to a chemical equilibrium. Then we linearise the model, compute the posterior mode, and move the current parameter set towards this mode, using a relaxation factor λ.

After running the algorithm for the first estimation step described above, we continue as follows:

1. Choose a relaxation parameter λ (e.g., $\lambda = 0.2$) and a convergence parameter ε (e.g., $\varepsilon = 10^{-5}$).
2. Compute a reduced stoichiometric matrix N_{R} and the corresponding link matrix L (see [5]) such that $N = L\ N_{\mathrm{R}}$ and N_{R} has full row rank.
3. Append additional empty columns to $R^{x}_{\theta^{\mathrm{kin}}}$ (for each enzyme and each external metabolite, in each steady state) to obtain the complete matrix R^{x}_{θ}. Append the prior means and the covariance matrix of the metabolic parameters θ^{met} to $\bar{\theta}^{\mathrm{kin}}_{(1)}$ and $C^{\mathrm{kin}}_{(1)}$, respectively. This yields $\bar{\theta}_{(1)}$ and $C_{(1)}$.
4. For each steady state m,
   - Parse the metabolic data, construct the data vectors $s^{*}_{(m)}$ (concentrations), $j^{*}_{(m)}$ (fluxes), and $E^{*}_{(m)}$ (enzyme concentrations). If data values are missing, the corresponding elements are not listed in the vectors. Construct the corresponding vectors of measurement noise levels $\sigma^{*}_{\mathrm{s(m)}}, \sigma^{*}_{\mathrm{j(m)}}, \sigma^{*}_{\mathrm{u(m)}}$.

- Determine which of the metabolites are described by fixed values in the model and have been measured in steady state m. Collect their measured concentrations in a vector $\tilde{s}^*_{(m)}$. Let $\tilde{N}_{(m)}$ denotes the corresponding stoichiometric matrix. Determine a left-kernel matrix $G_{(m)}$ of $\tilde{N}_{(m)}$. Compute the vector of conserved moieties $f_{(m)} = G_{(m)}\tilde{s}^*_{(m)}$
- Initialise the vector $\hat{s}_{(m)}$ of initial concentrations with a value of 1 for each element.

5. Collect the metabolic data $s^*_{(m)}, j^*_{(m)}, E^*_{(m)}$ for all steady states m in a vector y^* and construct the diagonal covariance matrix $C_{\rm y}$ for the measurement noise.

6. Initialise all model parameters (elements of $\hat{\theta}$) with 1.

7. Compute all steady state quantities $y(\hat{\theta})$ and the corresponding response coefficients matrix $R(\hat{\theta})$, based on the initial values $\hat{s}_{(m)}$ (computation see below).

8. Iterate until convergence:
   - Compute the posterior mode $\hat{\theta}^{\rm mode}$ of the current linearised model from eqn. (13). Compute the change $\Delta\hat{\theta} = \hat{\theta}^{\rm mode} - \hat{\theta}$ and update $\hat{\theta} \rightarrow \hat{\theta} + \lambda\ \Delta\hat{\theta}$.
   - For each steady state m, compute a change for the initial concentrations $\Delta\hat{s}_{(m)} = G^{\rm T}_{(m)} \left(G_{(m)}G^{\rm T}_{(m)}\right)^{-1} \left(f_{(m)} - G_{(m)}\hat{s}_{(\rm m)}\right)$ and update $\hat{s}_{(m)} \rightarrow \hat{s}_{(m)} + \lambda\ \Delta\hat{s}_{(m)}$. This improves the match to the experimental conserved moieties.
   - Compute again all steady state quantities $y(\hat{\theta})$ and the corresponding response coefficients matrix $R(\hat{\theta})$, based on the new parameters $\hat{\theta}$ and the new initial values $\hat{s}_{(m)}$. If no steady state is found, choose a smaller λ and repeat the last iteration step.

9. As convergence criterion, we require that
$$\max_k \left|\frac{\hat{\theta}_k^{\rm new} - \hat{\theta}_k^{\rm old}}{\hat{\theta}_k^{\rm new} + \hat{\theta}_k^{\rm old}}\right| < \varepsilon$$

10. Accept the final $\hat{\theta}$ as $\bar{\theta}_{(2)}$ and compute the corresponding covariance matrix $C_{(2)}$ from eqn. (13).

Calculation of steady state quantities

The computation of the steady state quantities works as follows:

1. For each steady state m:
   - Assume the system parameters $\hat{\theta}^{\rm kin}$, metabolic parameters $\theta^{\rm met}_{(m)}$, and initial concentrations $\hat{s}_{(m)}$.
   - Initialise the system with concentrations $\hat{s}_{(m)}$, find the nearest steady state $s_{(m)}$ fulfilling $0 = Nv(s_{(m)}, \theta^{\rm kin}, \theta^{\rm met}_{(m)})$, and compute the fluxes $j_{(m)} = v(s_{(m)}, \theta^{\rm kin}, \theta^{\rm met}_{(m)})$. Compute the enzyme concentrations $E_{(m)}$ directly from $\theta^{\rm met}_{(m)}$.
   - Compute the response matrices $R^{\rm S}_{(m)}$ and $R^{\rm J}_{(m)}$. The response matrix with respect to the logarithmic system parameters θ is computed as
$$R_{(m)} = \hat{R}_{(m)}\ {\rm diag}(x)\ R^x_\theta \qquad (3)$$
     from the matrix $\hat{R}_{(m)}$ of non-normalised response coefficients $\hat{R}_{im} = \partial y_i/\partial x_m$ (to be computed using using L, $N_{\rm R}$, and the elasticity matrices, see [5]).

2. Collect the concentration vectors $s_{(m)}$, the flux vectors $j_{(m)}$, and the enzyme concentrations $E_{(m)}$ in a vector y, and the corresponding response coefficients matrices in a response coefficient matrix R. Keep only the elements in y and rows in R for which data are available.

References

1. Hucka M, et al.: **The Systems Biology Markup Language (SBML): A Medium for Representation and Exchange of Biochemical Network Models**. *Bioinformatics* 2003, **19**(4):524–531.

2. Olivier B, Snoep J: **Web-based kinetic modelling using JWS Online**. *Bioinformatics* 2004, **20**(13):2143–2144.

3. Chassagnole C, Raïs B, Quentin E, Fell DA, Mazat J: **An integrated study of threonine-pathway enzyme kinetics in Escherichia coli**. *Biochem J* 2001, **356**:415–423.

4. Liebermeister W, Klipp E: **Bringing metabolic networks to life: convenience rate law and thermodynamic constraints**. *Theor Biol Med Mod*.

5. Reder C: **Metabolic control theory: a structural approach**. *J Theor Biol* 1988, **135**:175–201.
